# Extraction Methods for Brain Biopsy NMR Metabolomics: Balancing Metabolite Stability and Protein Precipitation

**DOI:** 10.3390/metabo14110609

**Published:** 2024-11-10

**Authors:** Wenzheng Xiong, Florian Zirpel, M. Zameel Cader, Daniel C. Anthony, Fay Probert

**Affiliations:** 1 Department of Chemistry, University of Oxford, Oxford OX1 3TA, UK; wenzheng.xiong@some.ox.ac.uk; 2 Department of Pharmacology, Medical Sciences Division, University of Oxford, Oxford OX1 3QT, UK; daniel.anthony@pharm.ox.ac.uk; 3 Nuffield Department of Clinical Neurosciences, University of Oxford, Oxford OX3 9DU, UK; florian.zirpel@ndcn.ox.ac.uk (F.Z.); zameel.cader@ndcn.ox.ac.uk (M.Z.C.)

**Keywords:** metabolomics, extraction, brain, NMR, metabolites

## Abstract

**Background/Objectives**: Metabolic profiling of tissue samples via liquid-state nuclear magnetic resonance (NMR) requires the extraction of polar metabolites in a suitable deuterated solvent. Such methods often prioritise metabolite recovery over protein removal due to the relatively low sensitivity of NMR metabolomics and the routine use of methods able to supress residual protein signals. However, residual protein may impact metabolite integrity and the metabolite stability after NMR sample preparation is often overlooked. This study aimed to investigate the effect of residual protein contamination in rodent brain extracts and identify a reproducible extraction method that optimises metabolite recovery while ensuring sample stability. **Methods**: The performance of acetonitrile/water (50–100% MeCN), methanol/water (50–100% MeOH), and methanol/water/chloroform (MeOH/H_2_O/CHCl_3_) were assessed for extraction efficiency, reproducibility, residual protein contamination, and metabolite stability up to eight hours post NMR sample preparation. **Results**: Aspartate and glutamate deuteration were observed in 50% MeCN, 50% MeOH, and 67% MeOH extractions along with the conversion of N-acetyl aspartate to aspartate and acetate in 50% MeCN and 50% MeOH extractions. Both observations correlated with residual protein contamination and, thus, are a result of inadequate protein precipitation, as confirmed by ultrafiltration. MeOH/H_2_O/CHCl_3_ extraction preserved the stability of these metabolites while maintaining good extraction efficiency and reproducibility. **Conclusions**: Thus, we recommend MeOH/H_2_O/CHCl_3_ extraction for untargeted brain NMR metabolic profiling due to its effective protein precipitation and reliable performance. Nonetheless, the performance of detecting metabolites prone to oxidation such as ascorbate and glutathione is not improved by this method.

## 1. Introduction

Metabolic profiling aims to measure the full complement of low-molecular-weight metabolites within biological samples, offering a biochemical snapshot that mirrors the current physiological condition of an organism [1]. The field of metabolomics, particularly in examining brain tissues, has evolved, allowing researchers to interrogate the biochemical shifts associated with varying physiological states and to understand better the downstream molecular mechanisms that contribute to the outcome of individual disease pathologies [2].

Nuclear magnetic resonance (NMR) and mass spectrometry (MS) are the primary analytical techniques in metabolomics research. Although NMR has limitations in sensitivity, detecting metabolites above micromolar levels, its advantages are significant. These include high reproducibility, intrinsic quantification capabilities, and a non-destructive analytical process, meaning the sample remains intact after data acquisition. Additionally, pulse programmes can be used to suppress chemical shifts arising from macromolecules, which greatly simplifies sample preparation [3]. Notably, NMR accounts for about 20% of all brain metabolomics research, as indicated by PubMed data showing 383 references for “metabolomics AND brain AND nuclear magnetic resonance NOT MRI NOT HRMAS” compared to 1600 for “metabolomics AND brain AND mass spectrometry NOT MSI” over the last two decades.

Metabolic profiling via liquid-state NMR requires the extraction of biological tissue samples, the concentration of metabolites to levels compatible with NMR detection, and the simultaneous removal of macromolecules in a manner that preserves metabolite stability. The selection of the extraction method, including the choice of extraction solvent, depends on factors such as the type of biological sample studied and the molecules of interest. Methanol/water and acetonitrile/water are commonly favoured for their efficient extraction of polar metabolites, precipitation of proteins, analytical compatibility, and ease of handling [4]. Additionally, methanol/water/chloroform is commonly used for simultaneous biphasic extraction of polar and non-polar metabolites and for improved protein precipitation, despite being more time-consuming [5].

The choice of extraction solvent can significantly impact reproducibility, metabolite recovery, and protein removal. For instance, methanol has emerged as the preferred extractant for plasma samples due to its superior performance in these areas compared to acetonitrile [6,7,8,9,10]. Similarly, different extraction methods have been evaluated in liver tissues, where acetonitrile/water demonstrated the highest metabolite yield but also recovered some macromolecules and lipids, whereas methanol/water/chloroform provides excellent deproteinisation while maintaining high yield and reproducibility [5]. In NMR analysis of brain extracts, acetonitrile/water also showed better yield compared to methanol/water and methanol/dichloromethane/water, while methanol/water showed lower reproducibility [11].

Due to the reduced sensitivity of NMR relative to MS along with the routine use of pulse programs, such as the Carr–Purcell–Meiboom–Gill sequence, which filter out macromolecule signals, extraction methods which prioritise metabolite recovery over deproteinisation are often favoured in NMR metabolomics studies. However, residual proteins in samples may still alter metabolic profiles either through enzymatic activity or binding to free metabolites. Indeed, it has been shown that brain homogenates, containing residual protein, undergo significant metabolic changes with prolonged incubation, whereas minimal changes occur in the aqueous phase of brain extracts subjected to the methanol/chloroform/water method after incubation [12]. Furthermore, Paskevich et al. discovered that after NMR sample preparation, the alpha-hydrogen (αH) of aspartate was replaced by deuterium from the NMR buffer, whereas deproteinated samples extracted with a methanol/water/chloroform mixture could prevent this replacement [13]. 

**Figure 1:**
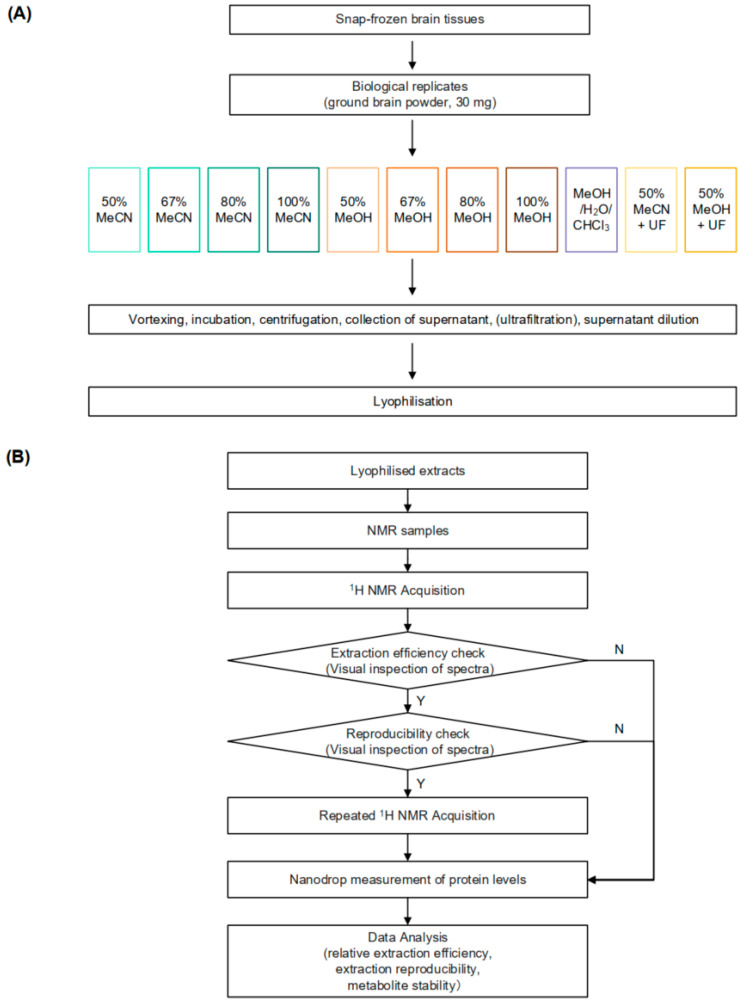
Workflow of sample preparation and NMR acquisition for metabolic profiling of brain tissues using various extraction methods. (**A**) Steps for brain metabolite extraction. (**B**) Flow of ^1^H NMR data acquisition and data analysis. The criteria for good extraction efficiency and reproducibility are defined as having a relative extraction efficiency greater than 0.7 and a median relative standard deviation (RSD) of less than 20%. UF, ultrafiltration. N, no. Y, yes.

This observation gives rise to the hypothesis that residual proteins in brain NMR samples can continue to alter the metabolome. While residual enzymatic activity is a well-documented issue in metabolomics, particularly for biofluids such as serum, plasma, and urine, where it has been extensively studied [14,15,16,17], these biofluids can often be analysed directly by NMR after being mixed with an appropriate buffer, without the need for an extraction step. In contrast, brain tissue samples require a more complex preparation process, including an extraction step, to obtain a liquid-state NMR spectrum. Prior to NMR analysis, mixing the lyophilised brain tissue extracts with the NMR buffer (e.g., deuterated phosphate buffer, pH 7.4) can potentially reactivate residual enzymes, leading to metabolite conversions. Despite the critical role of extraction in preparing brain tissues for NMR analysis, no research has systematically investigated the relationship between metabolite stability and residual protein levels across different extraction methods in rodent brain extracts. The study at hand aims to bridge this gap in knowledge by evaluating whether such alterations exist with commonly used extraction methods, understanding their impact, and identifying ways to control them. By minimising the effects of preanalytical variability, we can ensure that the measured metabolites accurately reflect the in vivo state, leading to improved interpretation. Specifically, we will assess the extraction performance of various solvents on mouse brain tissues, focusing on extraction efficiency, reproducibility, metabolite stability up to eight hours post NMR sample preparation, and residual protein concentrations (Figure 1). We hypothesise that adequate protein precipitation will preserve metabolite integrity during delays between sample preparation and analytical measurement, thereby providing more reliable NMR-based metabolic profiles.

For our evaluation, we included 11 extraction methods: acetonitrile (MeCN, 50%, 67%, 80%, 100%, *v*/*v*), methanol (MeOH, 50%, 67%, 80%, 100%, *v*/*v*), methanol/water/chloroform (MeOH/H_2_O/CHCl_3_, 2:1:2, *v*/*v*), 50% MeCN with ultrafiltration, and 50% MeOH with ultrafiltration. Acetonitrile and methanol were selected as the primary extraction solvents due to their ability to precipitate proteins, extract wide ranges of metabolites, and their widespread application in metabolomics [18]. Acetonitrile is relatively less polar than methanol, making it less soluble for proteins [19], while methanol is relatively more polar, thereby increasing solubility for polar metabolites [8]. The ratio of organic solvent to water is important for efficient protein removal [19]. We included varying concentrations of methanol and acetonitrile to identify a balanced ratio optimising both metabolite recovery and sufficient protein precipitation. In this study, 50% MeOH and 50% MeCN were used as negative controls due to their observed instability of the key metabolites. These methods provided a baseline for comparison, allowing us to evaluate the performance of more optimised extraction protocols in maintaining metabolite stability. The methanol/water/chloroform method, widely used in brain extraction since its description by Folch [20], was also included. Improved protein precipitation was expected as the decreased polarity of chloroform and the formation of two distinct phases of methanol/chloroform caused proteins to precipitate at the interface. It has been suggested that the addition of chloroform may be required for sufficient lipid removal from lipid-rich tissues such as the brain [5]. Finally, we included the use of ultrafiltration with a molecular weight cut-off (MWCO) filter as a positive control for protein removal.

## 2. Materials and Methods

### 2.1. Brain Tissues

Brain tissues were collected from C57BL/6/J mice, after the animals were terminally anesthetised with isoflurane and transcardially perfused with heparinised saline (5000 USP/L). Samples were stored at −80 °C until they were used for experimental purposes.

Snap-frozen brain tissues were pooled and homogenised using a pestle and mortar on dry ice, a widely recognised method in metabolomics for ensuring consistency across biological replicates [5,11,12,21,22]. A brain mass of 30 mg (resulting in 42 replicates with a mean of 30.6 ± 0.7 mg) was selected for further extraction based on a balance between metabolite detection sensitivity and tissue conservation [21].

### 2.2. Brain Metabolite Extraction

Detailed procedures of the 11 extraction protocols used were as follows. The protocols were adapted based on our previous experience and the existing literature [5,11,12,21,22]. 

#### 2.2.1. MeCN/H_2_O Extraction Protocol

For each sample, we added an ice-cold acetonitrile–water mixture, at a ratio of 8-to-1 (in microliters per milligram of sample), with concentrations ranging from 50% to 100% (*v*/*v*). This mixture was vortexed vigorously for 30 s to ensure thorough mixing. It was then incubated on ice for 15 min, followed by centrifugation at 16,000× *g* for 15 min at 4 °C. We collected 200 microliters of the clear supernatant from each sample and diluted it with H_2_O to adjust the final acetonitrile concentration to 40%, enabling it to be freeze-dried effectively. Subsequently, these diluted extracts were freeze-dried and stored at −80 °C until use in NMR spectroscopy.

#### 2.2.2. MeOH/H_2_O Extraction Protocol

For each sample, an ice-cold methanol–water mixture with varying concentrations (50%, 67%, 80%, and 100% *v*/*v*) was added at a ratio of 8 microliters per milligram of sample. Following the addition, the samples were vigorously mixed using a vortex mixer for 30 s, incubated on ice for 15 min, and then centrifuged at 16,000× *g* for 15 min at 4 °C. From each sample, 200 microliters of the supernatant were collected. The collected supernatants were further diluted with H_2_O to achieve a final methanol concentration of 20%. The resulting extracts were then freeze-dried and stored at −80 °C until use in NMR spectroscopy.

#### 2.2.3. MeOH/H_2_O/CHCl_3_ Extraction Protocol

For each sample, a volume of ice-cold 100% methanol (MeOH) was added at a ratio of 5.3 microliters per milligram of sample. The mixture was then subjected to 30 s of vigorous vortexing. Subsequently, a volume of ice-cold H_2_O equivalent to 2.7 times the sample mass (µL/mg) and a volume of ice-cold chloroform (CHCl_3_) equivalent to 5.3 times the sample mass (µL/mg) were added to each sample. The samples were mixed with a vortex for 30 s, then incubated on ice for 15 min. Following the incubation, the samples were centrifuged at 16,000× *g* for 15 min at 4 °C to facilitate phase separation. After centrifugation, 200 microliters of the aqueous phase (methanol/water phase) was collected from each sample. This collected phase was then diluted with H_2_O to adjust the final methanol concentration to 20%. The diluted extracts were freeze-dried and stored at −80 °C until use in NMR spectroscopy.

#### 2.2.4. MeCN/H_2_O and MeOH/H_2_O Extraction with Ultrafiltration Protocol

A total of 200 µL of MeCN/H_2_O or MeOH/H_2_O extract was filtered with 10 kD MWCO filters (Amicon^®^ Ultra 0.5mL centrifugal filters UFC501024, Merck KGaA, Darmstadt, Germany; 16,000× *g*, 15 min, 4 °C). Prior to the use, the filters were washed with 500 µL H_2_O (16,000× *g*, 30 min, 4 °C) to remove glycerol. Despite the wash, glycerol contamination persisted in ultrafiltration samples. Elusion (about 170 µL) was collected and subsequently diluted with H_2_O to create extracts with a final MeCN concentration of 40% or a final MeOH concentration of 20%, lyophilised, and stored at −80 °C until use in NMR spectroscopy. 

### 2.3. H NMR Analysis

Lyophilised extracts were resuspended with 550 µL 75 mM phosphate buffer D_2_O (pH 7.4) containing 32.2 µM sodium salt of 3-(trimethylsilyl) propionic acid-2,2,3,3-d_4_ (TSP) and transferred to a 5 mm borosilicate NMR tube (NORELL^®^, Inc., Morganton, NC, USA). Deuterated solvents (D_2_O) were used exclusively for preparing the NMR buffer to ensure accurate spectral acquisition and minimise water signal interference. For the extraction protocols, regular non-deuterated water (H_2_O) was used, as specified in the main text. NMR spectroscopy was performed using a 700-MHz Bruker AVIII spectrometer (Department of Chemistry, University of Oxford) operating at 16.4 T equipped with a ^1^H [^13^C/^15^N] TCI cryoprobe at 298 K. ^1^H NMR spectra were acquired by using the noesygppr1d pulse sequence (Bruker Corporation, Billerica, MA, USA, a one-dimensional nuclear Overhauser effect spectroscopy [NOESY] presaturation scheme) with the following parameters: td = 32,768, d1 = 2 s, p1 = 16.03 µs, ns = 64, aq = 1.46 s, sw = 16 ppm. The total acquisition time for the proposed NOESY spectra was approximately 12 min, consisting of 4 min for data collection and an additional 8 min allocated for sample loading, temperature equilibration, locking, shimming, and tuning. Repeated NMR measurements were conducted for samples that met both the extraction efficiency and reproducibility criteria (defined as extraction efficiency < 0.7 and/or median RSD > 20%), acquiring data at hourly intervals up to an 8-h delay while stored on the carousel at room temperature (Figure 1B).

### 2.4. Measurement of Protein Concentrations

The protein concentration of each NMR sample was determined using absorbance measurements at 205 nm with the NanoDrop™ One instrument (Thermo Fisher Scientific, Waltham, MA, USA).

### 2.5. NMR Data Pre-Processing

Resulting free induction decays (FIDs) were zero-filled by a factor of 2 and multiplied by an exponential function corresponding to 0.30 Hz line broadening prior to Fourier transformation. All spectra were phased, baseline corrected (using a 3rd degree polynomial), and chemical shifts referenced to the lactate resonance at δ   1.33 ppm in Topspin 4.1 (Bruker Corporation, Billerica, MA, USA) and exported to ACD/Labs Spectrus Processor Academic Edition 12.01 (Advanced Chemistry Development, Inc., Toronto, ON, Canada).

A semi-targeted analysis approach was employed by manually dividing each NMR spectrum into 86 spectral buckets for integration, excluding noise and water signals, to minimise contributions from varying protein levels across different methods. For samples prepared using MWCO filters, contaminant signals arising from glycerol were also removed. NMR buckets and corresponding assignments are listed in Table S1 (Figure S1, Table S1). Metabolite assignment was performed in reference to the literature values [11,12,13,23] via metabolite spiking and 2D total correlation spectroscopy (TOCSY) experiments. The absolute integral values were subject to the main statistical analysis unless stated otherwise. Integral values were mean centred and scaled to unit variance prior to principal component analysis.

### 2.6. Data Analysis

Analysis was performed in the R software 4.1.2 (R foundation for statistical computing, Vienna, Austria). The ggplot2 (version 3.4.2), pheatmap (version 1.0.12), ropls (version 1.26.4), and metabom8 (version 1.0.0) packages were used for the generation of the plots [24,25,26,27]. The relative extraction efficiency was determined by calculating the mean total intensity of NMR integrals, normalised to that of the 50% MeCN group. The extraction reproducibility was assessed using the median of the relative standard deviation (RSD) of the replicates for each metabolite resonance. The stability of each spectral bucket was determined by calculating the average percentage change over time up to 8 h for each bucket across three replicates. The one-way analysis of variance (ANOVA) was used to identify significant differences between the means of groups. Two-tailed *p*-values ≤ 0.05 were considered statistically significant.

## 3. Results and Discussion

### 3.1. Relative Extraction Efficiency

**Figure 2:**
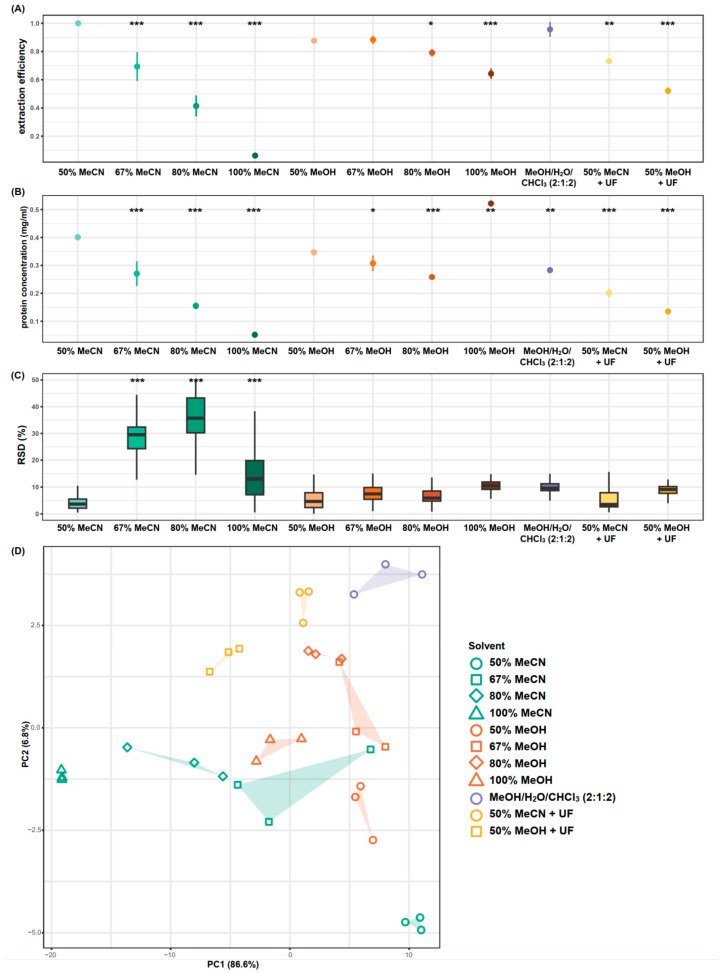
Efficiency and reproducibility of the extraction methods tested. (**A**) Relative extraction efficiency, expressed as the mean ± SEM, calculated as the sum of integrals normalised to that of the 50% MeCN group. (**B**) Protein levels of the NMR samples derived from different brain extracts, expressed as the mean ± SEM. (**C**) Extraction reproducibility, presented in boxplots, as determined by the relative spectral standard deviation across each of the 86 spectral buckets. (**D**) PCA scores plot of the brain metabolic profiles from different extraction methods at 0 h delay in NMR measurement. A smaller spread of the polygon indicates better reproducibility. Results of one-way ANOVA with Dunnett’s test for multiple comparisons are reported in reference to the 50% MeCN group. UF, ultrafiltration. * *p* < 0.05, ** *p* < 0.01, *** *p* < 0.001.

Among the 11 methods examined, 50% MeCN, MeOH/H_2_O/CHCl_3_, 50% MeOH, and 67% MeOH showed comparably high extraction efficiency (defined as the total sum of integrals of the 86 spectral buckets measured, normalised to that of the 50% MeCN group), with mean values of 1 ± 0.01, 0.96 ± 0.05, 0.88 ± 0.01, and 0.88 ± 0.03, respectively (*p*-value > 0.05, one-way ANOVA) (Figure 2A). The remaining nine methods demonstrated lower extraction efficiencies to varying degrees compared to 50% MeCN (*p*-values < 0.05, one-way ANOVA).

The increase in MeCN and MeOH concentrations led to a decrease in extraction efficiency, with MeCN showing a more pronounced decline. This trend was expected since hydrophilic metabolites had limited solubility in organic solvents such as MeCN, and to a lesser extent, MeOH, and in addition, it is supported by a previous serum extraction study [8]. Furthermore, ultrafiltration with MWCO filters resulted in a 27% reduction in extraction efficiency compared to unfiltered 50% MeCN and a 41% reduction compared to unfiltered 50% MeOH (*p*-values < 0.001, one-way ANOVA). In addition, residual glycerol arising from the MWCO filters results in large contaminant signals which obscure metabolite resonances, such as the ones for glycine and glutamine and myo-inositol. Representative NMR spectra for each extraction method are shown in Figure S2.

### 3.2. Protein Levels

No significant difference was observed in the protein levels of samples extracted with 50% MeCN and 50% MeOH (*p*-value > 0.05, one-way ANOVA, *n* = 3 per group). Increasing concentrations of solvent resulted in lower protein concentrations (*p*-values < 0.001 and <0.01 for MeCN and MeOH, respectively, one-way ANOVA), with the exception of samples extracted with 100% MeOH which showed higher protein levels (*p*-value < 0.001, one-way ANOVA). Using MeOH/H_2_O/CHCl_3_ and ultrafiltration also resulted in lower sample protein concentrations compared to 50% MeCN or 50% MeOH (*p*-value < 0.01, one-way ANOVA), with ultrafiltration yielding the lowest protein concentrations among these three methods (Figure 2B).

### 3.3. Extraction Reproducibility

The 67% MeCN, 80% MeCN and 100% MeCN extraction methods demonstrated significantly poorer reproducibility compared to the other extraction methods, as determined by RSD across all 86 spectral buckets measured (*p*-values < 0.001, one-way ANOVA) (Figure 2C). No significant differences were observed between the remaining extraction methods, with median RSDs ranging from 3.54% (interquartile range [IQR] 5.55%) to 10.56% (IQR 2.81%). PCA illustrates the clustering and dispersion patterns of each extraction method, revealing the similarities among and within each method (Figure 2D). Most methods resulted in distinct clusters in the PCA scores plot, highlighting global metabolite differences across these methods. Methods with lower extraction efficiency tended to cluster towards the left side of the scores plot, indicating lower metabolite yield. For instance, as the concentration of MeCN increased, samples tended to cluster further to the left. Conversely, methods with better protein removal, as identified by lower sample protein concentrations, tended to cluster towards the top of the plot.

Five extraction methods, specifically 67% MeCN, 80% MeCN, 100% MeCN, 100% MeOH, and 50% MeOH with ultrafiltration, demonstrated poor extraction efficiency or reproducibility (as defined by extraction efficiencies < 0.7 and/or median RSD > 20%). As a result, these methods were excluded from further metabolite stability testing.

### 3.4. Metabolite Stability

**Figure 3:**
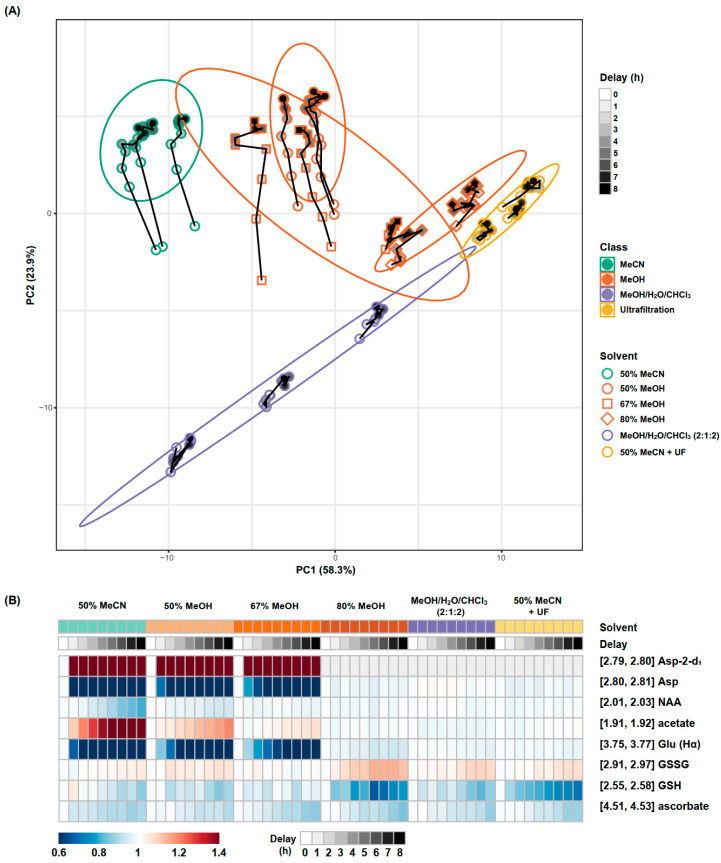
Metabolite extract stability assessment for tested methods. (**A**) PCA scores plot demonstrating the temporal metabolome changes after NMR sample preparation. The ellipse indicates the 95% confidence interval for a multivariate distribution for each extraction method. The trajectory lines connect data points at different delay times from the same sample. (**B**) Heatmap depicting percentage changes, relative to the 0 h timepoint, in unstable metabolites for each extraction method. No aspartate-2-d_1_ signals (grey) were observed in the 80% MeOH, MeOH/H_2_O/CHCl_3_ (2:1:2), and 50% MeCN with ultrafiltration groups. UF, ultrafiltration. Asp, aspartate. Glu, glutamate. NAA, N-acetyl aspartate. GSH and GSSG, reduced form and oxidised form of glutathione, respectively.

Metabolite stability was assessed at hourly intervals up to 8 h following sample preparation for the six extraction methods selected in the previous section. Among the six extraction methods that demonstrated adequate efficiency and reproducibility, only two low-concentration metabolites, CoA and adenosine, were undetectable in the 80% MeOH, MeOH/H_2_O/CHCl_3_, and 50% MeCN + UF methods (Figure S3). The trajectory of each extract over time was visualised using a PCA scores plot (Figure 3A). The 50% MeCN, 50% MeOH, and 67% MeOH samples displayed significant drift along the second principal component (PC2). Inspection of the loadings revealed that multiple aspartate signals (2.65–2.84 ppm) and glutamate signals (3.75–3.77 ppm) drove the variability of PC2 (Figure S4). The changes that occur over time are reproducible in each sample, indicating the alteration in metabolic profile is time-dependent; for example, the 8 h samples were equivalent to each other in each extraction method. Table 1 summarises the stability of the whole spectrum over time for each method. The 50% MeCN, 50% MeOH, and 67% MeOH extraction methods resulted in a larger number of metabolite resonances with an RSD greater than 30% after eight hours. A heatmap illustrates the changes in each metabolite resonance over time for the extraction methods (Figure S3), with the most variable metabolites (aspartate, glutamate, N-acetyl aspartate [NAA], acetate, glutathione, and ascorbate) summarised in Figure 3B. 

#### 3.4.1. Aspartate and Glutamate

**Figure 4:**
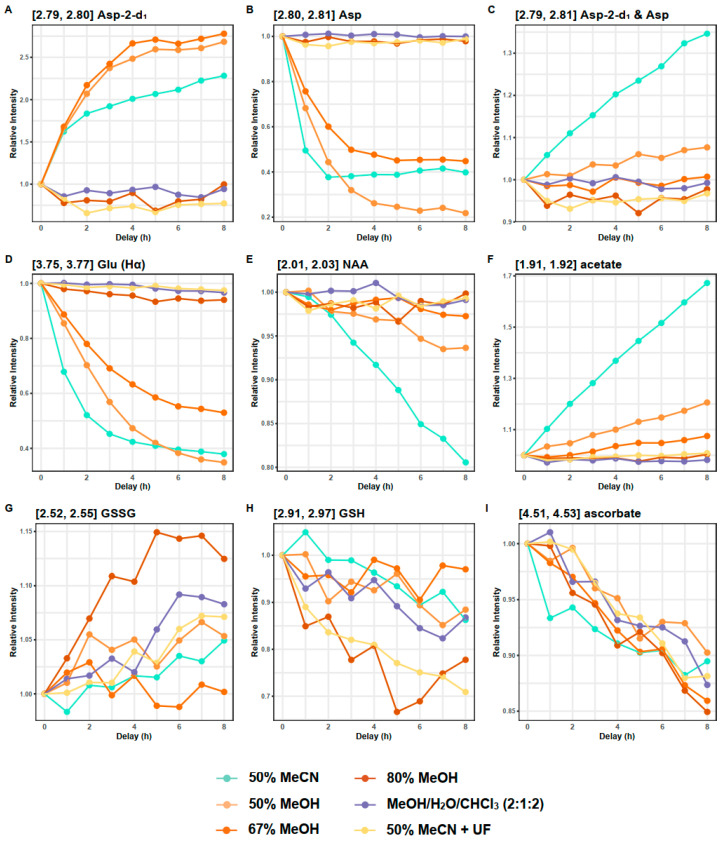
Changes in unstable metabolites across different extracts over time post sample preparation. Each data point refers to the mean of three replicates. Values were normalised to the 0 h delay group within each extraction method. UF, ultrafiltration. Asp, aspartate. Glu, glutamate. NAA, N-acetyl aspartate. GSH and GSSG, reduced form and oxidised form of glutathione, respectively.

The instability of aspartate signals was observed in samples extracted with 50% MeCN, 50% MeOH, and 67% MeOH, indicated by a decrease in the doublet of doublet (dd) signals and the generation of nearby doublets signals at 2.82 and 2.68 ppm (*p*-values < 0.05, one-way ANOVA) (Figure 4 and Figure S3, Table S1). This change was most rapid within the initial hour after NMR sample preparation, with the original aspartate signals disappearing after two hours in samples extracted with 50% MeCN and after four hours in samples extracted with 50% and 67% MeOH (Figure S5). 

Similarly, the glutamate multiplets, observed at 2.12 ppm and 2.35 ppm, exhibited a reduction in splitting in samples extracted with solvent mixtures of 50% MeCN, 50% MeOH, and 67% MeOH (*p*-values < 0.05, one-way ANOVA) (Figure S3). The overall integrals of these multiplets remained unchanged. Additionally, a considerable reduction was observed in the glutamate dd at 3.76 ppm in samples extracted using these methods (Figure 4). The glutamate dd was completely diminished after four hours in samples extracted with 50% MeCN (Figure S5).

The observed instability in the resonances of aspartate is attributed to the deuteration of aspartate, a phenomenon previously documented by Paskevich et al. [13]. This involves the replacement of the proton at the α-carbon (αH) of aspartate with deuterium, converting aspartate into aspartate-2-d_1_. Consequently, it induces a change in the multiplicities of the βCH_2_-group in aspartate, transforming the dd from the ABX spin system to AB doublets [13].

Here, similar to aspartate, we observed deuteration of glutamate. Substitution of the glutamate αH with deuterium would result in the disappearance of the resonance at 3.76 ppm and induce changes in the multiplicities of the βCH_2_-group without altering the overall integrals.

The deuteration of aspartate and glutamate may be derived from aspartate transaminase (AST)-mediated metabolism. AST catalyses the interconversion of aspartate and α-ketoglutarate to oxaloacetate and glutamate, during which the H2 hydrogens of aspartate and glutamate undergo exchange with those of water (D_2_O in the NMR buffer) [28], leading to changes in the observed splitting patterns of NMR resonances. This suggests that AST may have remained active and did not fully precipitate during the extraction process, as supported by the high levels of remaining protein with these methods. While the proposed residual AST activity requires confirmation by independent experiments, our results confirmed that the deuteration is due to residual proteins, as ultrafiltration prevents the detection of any degree of deuteration.

#### 3.4.2. N-Acetyl Aspartate and Acetate

A reduction of N-acetyl aspartate (NAA) was observed in samples extracted with 50% MeCN, and to a lesser extent in those extracted with 50% MeOH (Figure 4 and Figure S3, Table S1, *p*-values < 0.05, one-way ANOVA). Concurrently, acetate levels increased (*r* = −0.94, *p*-value < 0.001, Pearson correlation, Figure S6) and aspartate-2-d_1_ levels continued to rise even after the original aspartate signals had vanished (*r* = −0.82, *p*-value < 0.001, Pearson correlation, Figure S6). These observations suggest a conversion of NAA into aspartate and acetate. This conversion is further evidenced by the negative correlation between aspartate and aspartate-2-d_1_ at 0–3 h (*r* = −0.94, *p*-value < 0.001, Pearson correlation) and their positive correlation at 4–8 h (*r* = 0.76, *p*-value < 0.01, Pearson correlation). Additionally, combining the aspartate resonance and the aspartate-2-d_1_ resonance showed a continuous increase over time in the samples extracted with 50% MeCN and 50% MeOH, while this remains unchanged in the 67% MeOH samples. This suggests the deuteration of aspartate and glutamate, and conversion of NAA to acetate and aspartate, represent distinct reactions.

In the brain, aspartoacylase (ASPA) catalyses the hydrolysis of NAA to produce acetate and aspartate [29]. Although we did not perform independent experiments to directly confirm the presence of ASPA in the extracts, the observed conversion patterns align with known ASPA activity. Therefore, it is likely that some residual ASPA retained bioactivity in the NMR samples of the brain extracts from 50% MeCN and, to a lesser extent, 50% MeOH, as supported by higher residual protein levels in these samples. The conversion of NAA to acetate and aspartate was reduced and eventually absent as MeOH concentration increased. Despite the presence of residual ASPA remaining speculative, the observed conversion of NAA to aspartate and acetate is protein dependent. The addition of CHCl_3_ and the use of ultrafiltration further enhanced protein precipitation with these methods and prevented such interconversion.

#### 3.4.3. Glutathione

Oxidation of glutathione (GSH) was observed in all samples (*p*-values < 0.05, one-way ANOVA) with the exception of samples extracted with 67% MeOH (*p*-value > 0.05, one-way ANOVA). A significant negative correlation was observed between GSH and glutathione disulphide (GSSG) over time (*r* = −0.83, *p*-value < 0.001, Pearson correlation) (Figure 4, Figures S3 and S5). This was most evident in samples extracted with 80% MeOH, where a 22% ± 1% reduction in GSH and a 12% ± 6% increase in GSSG was observed after eight hours. GSH oxidation was unrelated to the residual protein content within samples (as these changes still occurred in samples subjected to ultrafiltration) suggesting that spontaneous oxidation outweighs any contribution from glutathione reductase. This aligns with the unstable nature of glutathione and previous findings in NMR-based blood metabolomics, indicating that degassed solvents or derivatising agents are required for accurate GSH measurement [30].

#### 3.4.4. Ascorbate

For all extraction methods, ascorbate concentrations showed a gradual decrease over the eight-hour period, resulting in a cumulative reduction of 10–15% across all the methods after eight hours (*p*-values < 0.05, one-way ANOVA) (Figure 4 and Figure S5). The instability observed in ascorbate did not appear to be related to protein levels, as it still occurred in samples subjected to ultrafiltration. Additionally, a gradual reduction of ascorbic acid was observed in its standard solution in the NMR buffer (Figure S7). The spontaneous oxidation of ascorbate aligns with its well-known antioxidant properties and has been reported in previous studies [31].

#### 3.4.5. Macromolecules

In line with the suboptimal deproteinisation of 50% MeCN-extracted samples, broad macromolecule resonances were observed within the spectral regions of 0.8–1.0 ppm, 1.2–1.5 ppm, and 1.6–1.8 ppm. In contrast, the other five extraction methods, including 50% MeCN extraction with ultrafiltration, displayed a flat baseline in these regions (Figure S8), which is in agreement with previous reports [5,10].

Interestingly, while no broad macromolecule signals were detectable in samples extracted with 80% MeOH at 0 h, consistent with improved deproteinisation, two out of three replicates showed the appearance of increasingly broad signals over time at 0.85–0.90 ppm and 1.25–1.35 ppm (Figure S9). This led to increased leucine signals at 0.95–0.98 ppm over time (*p*-value < 0.001, one-way ANOVA), resulting in a cumulative increase of 13% after eight hours. Moreover, the lactate doublets at 1.33 ppm also had an increase of 9% after eight hours due to the increasing broad signals underneath it (*p*-value < 0.05, one-way ANOVA). These signals may arise due to the aggregation of residual protein in the sample over time. In contrast, 50% MeCN with ultrafiltration and MeOH/H_2_O/CHCl_3_ extraction resulted in NMR spectra devoid of any broad signals from macromolecules, providing optimal baselines for analysis. 

### 3.5. Summary and Recommendations

Among the tested methods, five methods (67% MeCN, 80% MeCN, 100% MeCN, 100% MeOH, and 50% MeOH with ultrafiltration) showed inadequate extraction efficiency and/or poor reproducibility, making them unsuitable for NMR brain metabolomics. The performance of the remaining six methods is summarised in Table 2, including relative extraction efficiency, reproducibility, protein levels, and metabolite stability.

Three out of the remaining six methods (50% MeCN, 50% MeOH, and 67% MeOH) demonstrated good extraction efficiency and reproducibility, but the deuteration of aspartate and glutamate resonances was observed, possibly due to the inadequate denaturation/precipitation of AST. This highlights the importance of considering the effects of deuterated solvents and care should be taken to ensure that the integrals of both the protonated and deuterated resonances are combined to avoid spurious results. Furthermore, 50% MeCN and 50% MeOH were found to induce NAA conversion into aspartate and acetate, potentially linked to the insufficient denaturation of ASPA which poses a risk of false discoveries, particularly in the following scenarios: (1) during prolonged room temperature exposure; (2) during lengthy experiments with many samples, leading to long experimental durations; (3) the analysis of tissues where ASPA, potentially responsible for NAA conversion, is known to express; or (4) for the study of systems where the expression of ASPA is essential to the experimental readout. Therefore, these methods are not advised for untargeted metabolomics when these unstable signals are included in the data analysis. Our results highlight the importance of optimising the deproteinisation of tissues where residual enzymes could affect sample stability (including deuteration) or where protein expression levels vary, even when pulse programs to suppress residual macromolecules are to be used.

Increasing the MeOH ratio to 80% increased protein precipitation ability, which sufficiently denatured residual proteins, but at the expense of decreased extraction efficiency. However, increasing broad signals over time led to an overestimation of lactate and leucine concentrations. This phenomenon was not observed in samples subjected to ultrafiltration or MeOH/H_2_O/CHCl_3_ extraction, likely indicating that the broad signals arise from incomplete protein precipitation. While this method offers simplicity and is well-suited for high-throughput studies, there is a risk of temporal inflation in the resonances of lactate and leucine.

Ultrafiltration, serving as a positive control for protein removal, reduced protein content by half to a 0.2 ± 0.02 mg/mL, as evidenced by the absence of broad macromolecule resonances up to eight hours post sample preparation. Moreover, ultrafiltration preserved the stability of aspartate, glutamate, NAA, and acetate, thus confirming that their instability originated from residual proteins. However, ultrafiltration led to a 27% reduction in overall metabolite abundance compared to the 50% MeCN extraction method. Additionally, glycerol contaminants from the filter masked the detection of glycine, certain myo-inositol, and glutamine resonances in the NMR spectrum. Consequently, ultrafiltration methods do not appear suitable for NMR analysis.

MeOH/H_2_O/CHCl_3_ extraction resulted in improved deproteinisation, while maintaining adequate extraction efficiency and reproducibility in agreement with previous studies [5,11,32]. Therefore, MeOH/H_2_O/CHCl_3_ extraction is recommended for untargeted brain NMR metabolic profiling over other tested methods. One disadvantage of MeOH/H_2_O/CHCl_3_ extraction is that it requires more time and careful handling compared to single-phase extraction methods, particularly during phase transfer, in order to mitigate variability. Moreover, this method does not address the observed decrease in ascorbate and oxidation of GSH. To maintain changes within 10% for glutathione and ascorbate, it is advisable to acquire spectra within 4 h of sample preparation. For researchers requiring higher accuracy for these metabolites, further optimisation of extraction methods may be necessary. This could include the addition of antioxidants, or derivatising agents, pH adjustments, the use of degassed solvents, and/or temperature control to reduce the risks of metabolite oxidation [12,30,31,33].

While recommending specific extraction protocols, we strongly advise conducting trials of NMR spectroscopy and analysis on biological replicates using the method of choice before progressing to studying valuable samples. Absolute integral values were used in data analysis throughout this study to investigate preanalytical impact, while sum normalization is typically used in metabolomic studies to mitigate the impact of concentration variations resulting from mass differences or pipetting errors (Figure S10). This study evaluated metabolite integrity at room temperature following NMR sample preparation. Not all NMR spectrometers are equipped with autosamplers that can maintain refrigerated conditions, and preparing and loading samples individually to mitigate enzymatic activity would be labour-intensive and inefficient. This limitation highlights the importance of effective deproteinisation during sample preparation to ensure metabolite stability. When feasible, using autosamplers with refrigeration capabilities is recommended, as this can further mitigate metabolite alterations and enhance the reliability of metabolomics analysis. The focus of this study was on the extraction and stability of polar metabolites, as NMR metabolomics is well-suited for aqueous phase extractions. Although the brain is a lipid-rich organ, lipidomics is better addressed using mass spectrometry, and thus, lipid extraction was not within the scope of this NMR-based study. We recommend that future studies incorporate lipidomic analyses for a more comprehensive metabolic profile.

Our findings are specific to brain tissue, which has unique biochemical characteristics, such as a high lipid content and distinct protein compositions. While the addition of chloroform appears necessary for effective protein precipitation in brain tissue, the requirements may differ for other tissues. The most efficient protein removal method may not always be optimal in terms of time and metabolite recovery. For instance, tissues that lack significant amounts of these enzymes might achieve sufficient protein removal with simpler single-phase extractions, such as 50% MeOH or 50% MeCN. These simpler methods could offer benefits in terms of reproducibility and ease of use, particularly for high-throughput workflows. Future studies would be valuable to explore and optimise extraction protocols tailored to the unique biochemical properties of different tissues.

Our study highlights the importance of optimising extraction methods for brain NMR metabolomics, particularly in balancing metabolite stability (including that affected by deuteration) and effective protein precipitation. While this study used brain tissues from rodent models, these techniques could be similarly applied to human brain biopsies, where the amount of tissue available is also limited. This approach has direct relevance to both animal studies and potential clinical applications in human tissue analysis. Future research should explore the applicability of these protocols to human samples, particularly in clinical settings where precise metabolic profiling is crucial for diagnosis and treatment monitoring. In such cases, while histology alone may not provide a definitive diagnosis, metabolic profiling could offer valuable complementary insights.

## 4. Conclusions

This study is the first systematic investigation into the effect of residual protein contamination on metabolite stability following NMR sample preparation in brain tissues. The findings underscore the crucial role of protein precipitation in maintaining metabolite integrity, particularly for key metabolites such as aspartate, glutamate, and N-acetyl aspartate. Our results demonstrate that insufficient protein precipitation in commonly used extraction solvents (50% MeCN, 50% MeOH, and 67% MeOH) leads to metabolite instability (including that affected by deuteration), which can markedly compromise the reliability of NMR-based metabolomics studies. These results highlight the importance of optimising extraction methods for effective deproteinisation to ensure the stability of NMR spectra—a factor often overlooked due to the tolerance of NMR instrumentation to residual protein contamination.

By identifying MeOH/H_2_O/CHCl_3_ extraction as a reproducible method for enhancing metabolite stability, we provide a valuable tool for brain metabolomics research, which can be readily applied to optimise sample preparation protocols and improve the accuracy of metabolomic profiling. Although MeOH/H_2_O/CHCl_3_ extraction was effective in preventing protein-related instabilities, further optimisation is required to improve the stability of oxidation-sensitive metabolites. Future research should investigate the use of antioxidants, degassed solvents, or other stabilising agents to mitigate oxidation during the extraction process, as this remains a limitation of the present study.

## Figures and Tables

**Table 1:** Reproducibility of metabolite resonances over an 8 h delay for NMR analysis for each extraction method.

RSD (%)	Number of Variables (%)					
	50% MeCN	50% MeOH	67% MeOH	80% MeOH	MeOH/ H_2_O/CHCl_3_ (2:1:2)	50% MeCN + UF
0–15	70 (82)	68 (81)	60 (71)	73 (90)	76 (94)	77 (95)
15–30	9 (11)	6 (7)	11 (13)	8 (10)	5 (6)	4 (5)
>30	6 (7)	10 (12)	13 (15)	0 (0)	0 (0)	0 (0)

RSD, relative standard deviation. RSD was calculated over nine timepoints (0–8 h delay) across three replicates for each variable. UF, ultrafiltration.

**Table 2:** Summary of the six brain metabolite extraction methods in terms of extraction efficiency (mean ± SE), reproducibility (median, [IQR]), protein levels (mean ± SE), and metabolite stability.

Extraction Method	Relative Extraction Efficiency (Mean ± SE)	Reproducibility, RSD (%, Median [IQR])	Protein Levels (mg/mL, Mean ± SE)	Asp and Glu Deuteration	NAA Conversion into Aspartate and Acetate	GSH Oxidation to GSSG	Ascorbic Acid Reduction	Macromolecule Signals
50% MeCN	1 ± 0.01	3.68 (3.49)	0.4 ± 0.01	++	++	+	+	+
50% MeOH	0.88 ± 0.01	4.66 (5.67)	0.35 ± 0.01	++	+	+	+	-
67% MeOH	0.88 ± 0.03	7.48 (4.49)	0.31 ± 0.03	++	-*	-	+	-
80% MeOH	0.79 ± 0.03	5.93 (3.85)	0.26 ± 0.01	-	-	++	+	+
MeOH/H_2_O/ CHCl_3_ (2:1:2)	0.96 ± 0.05	9.62 (2.66)	0.3 ± 0.01	-	-	+	+	-
50% MeCN + UF	0.73 ± 0.01	3.54 (5.55)	0.2 ± 0.02	-	-	+	+	-

-: no. -*: marginally. +: yes. ++: yes, and to a greater extent. SE, standard error. RSD, relative standard deviation. IQR, interquartile range. Asp, aspartate. Glu, glutamate. NAA, N-acetyl aspartate. GSH and GSSG, reduced form and oxidised form of glutathione, respectively. UF, ultrafiltration.

## Data Availability

The original data presented in this study are openly available in the Oxford University Research Archive at http://dx.doi.org/10.5287/ora-degojb2kw.

## Supplementary Materials

The following supporting information can be downloaded at: https://www.mdpi.com/article/10.3390/metabo14110609/s1, Figure S1: Metabolite assignments in a representative NMR spectrum from a brain Sample Extracted with 50% MeCN. Figure S2: Overview of representative NMR spectra for each extraction method. Figure S3: Heatmap of NMR resonance percentage changes for metabolites across extraction methods. Figure S4: Loadings plot of PCA (corresponding to scores plot on Figure 2D) highlighting the contributions of Asp-2-d1, Asp, and Glu to variation in PC2. Figure S5: NMR spectra illustrating changes in unstable resonances over time delays in NMR measurement in a 50% MeCN brain sample. Figure S6: Pearson correlation of unstable metabolites in 50% MeCN samples. Pearson correlation coefficient r is visualised with different sized circles in the upper triangular of the correlation matrix, and the r values are shown in the lower triangular. *p* values were adjusted with the false discovery rate. The plot includes only significant correlations. (A) 0–8-h samples. (B) 0–3-h samples (C) 4–8-h samples. Figure S7: NMR spectra illustrating the reduction in ascorbate signals over time in an ascorbic acid standard sample (in phosphate buffer pH = 7.4) during NMR Measurement. Figure S8: Enhanced baseline uniformity at 0.8–1.0 ppm, 1.2–1.5 ppm, 1.6–1.8 ppm in 50% MeCN samples with ultrafiltration (yellow) in contrast to 50% MeCN samples (green). Figure S9: NMR spectra illustrating the increasing broad signals over time at 0.85–0.90 ppm and 1.25–1.35 ppm in an 80%MeOH brain extract during NMR measurement. Figure S10: Sum normalised method reduced the impact of variations in overall sample concentration. This is compared to Figure 2D and Figure 3A, where absolute values were used for PCA. (A) PCA scores plot of the brain metabolic profiles from different extraction methods with no delay in NMR measurement. (B) PCA scores plot of brain metabolic profiles from six extraction methods over a time range from 0 to 8 h of delay in NMR measurement. Table S1: Mean ± standard error of metabolite resonance integrals across groups at different NMR measurement delays. One-way ANOVA was performed to assess significant differences between group means.
