# Supplementary material for: Extraction Methods for Brain Biopsy NMR Metabolomics: Balancing Metabolite Stability and Protein Precipitation
Source: Metabolites. 2024 Nov 10;14(11):609. doi: 10.3390/metabo14110609 (PMC11596573; doi:10.3390/metabo14110609)
Supplement: Supplementary file 1 [file metabolites-14-00609-s001.zip › Merged SI Figures.pdf]

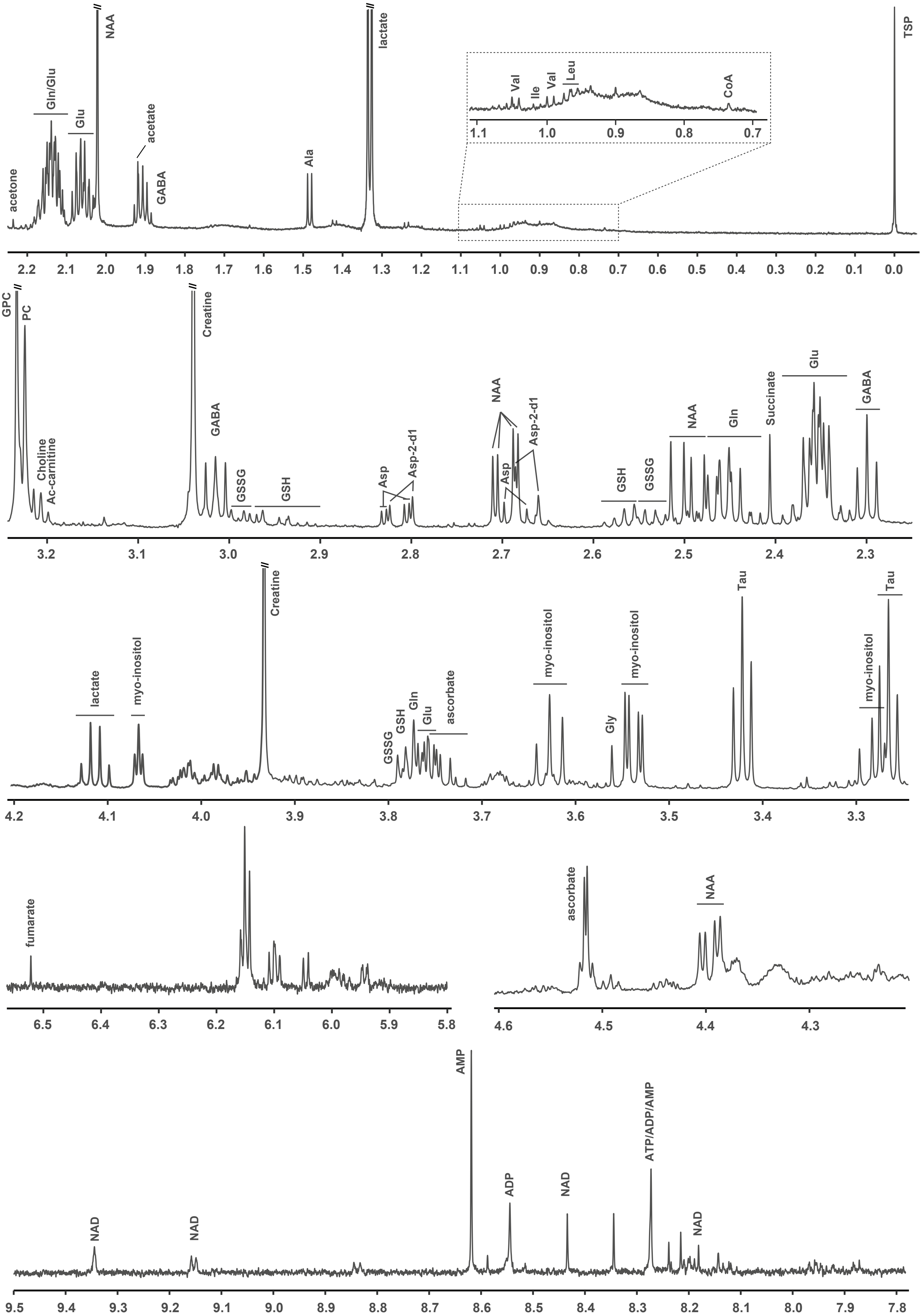

Figure S1. Metabolite assignments in a representative NMR spectrum from a brain Sample Extracted with 50% MeCN.

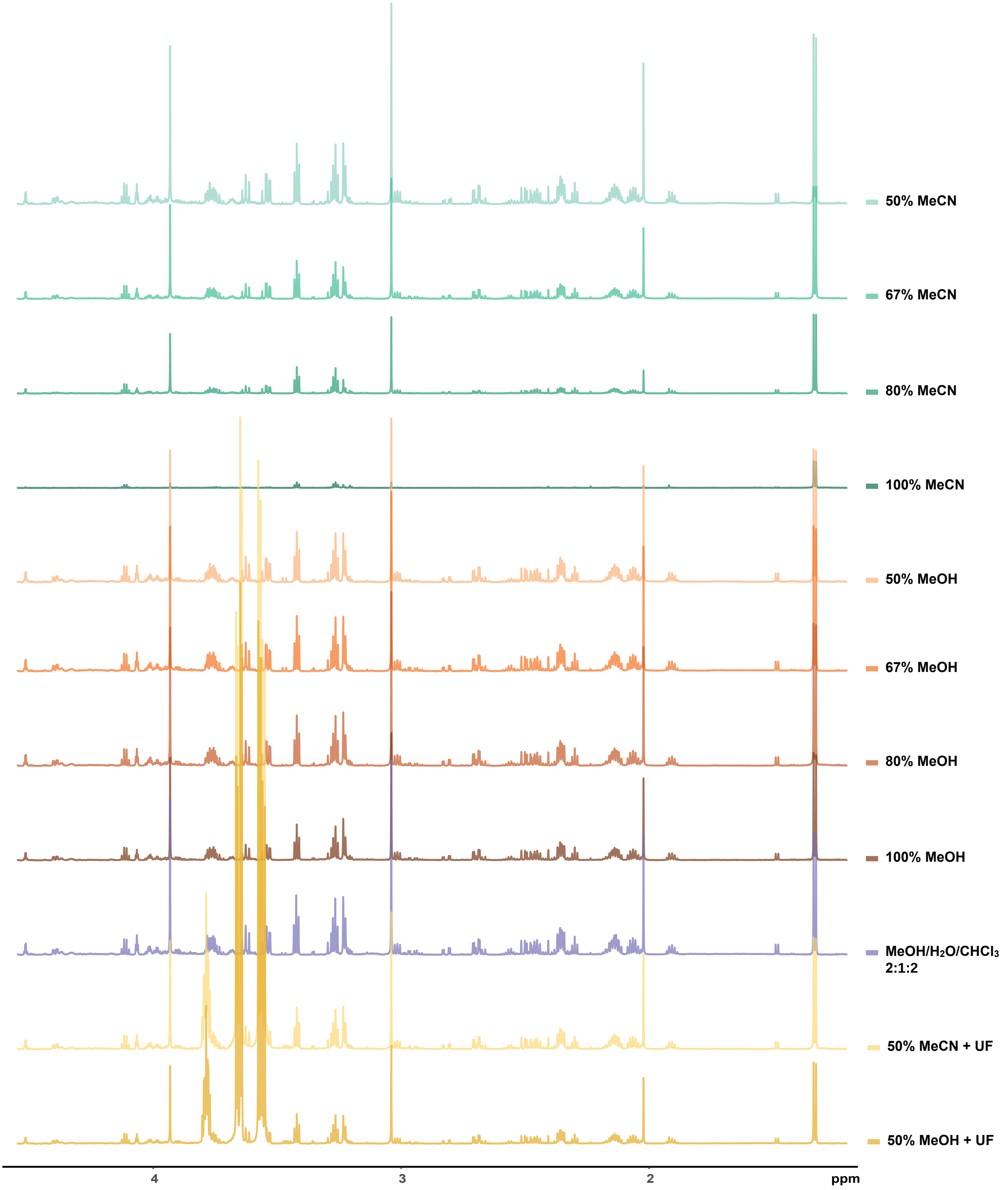
50% MeCN
67% MeCN
80% MeCN
100% MeCN
50% MeOH
67% MeOH
80% MeOH
100% MeOH
MeOH/H$_2$O/CHCl$_3$ 2:1:2
50% MeCN + UF
50% MeOH + UF
4
3
2
ppm

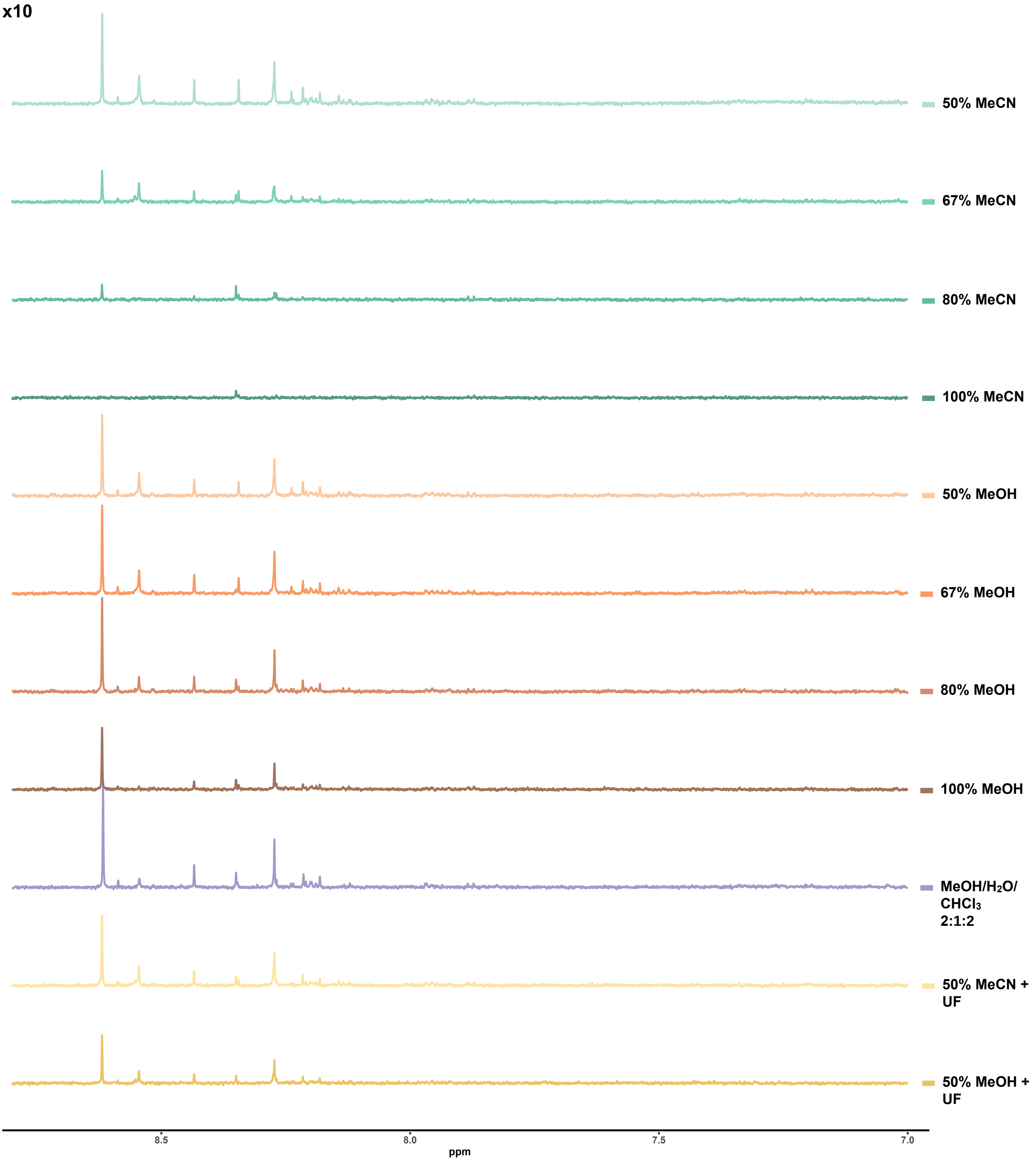

Figure S2. Overview of representative NMR spectra for each extraction method.

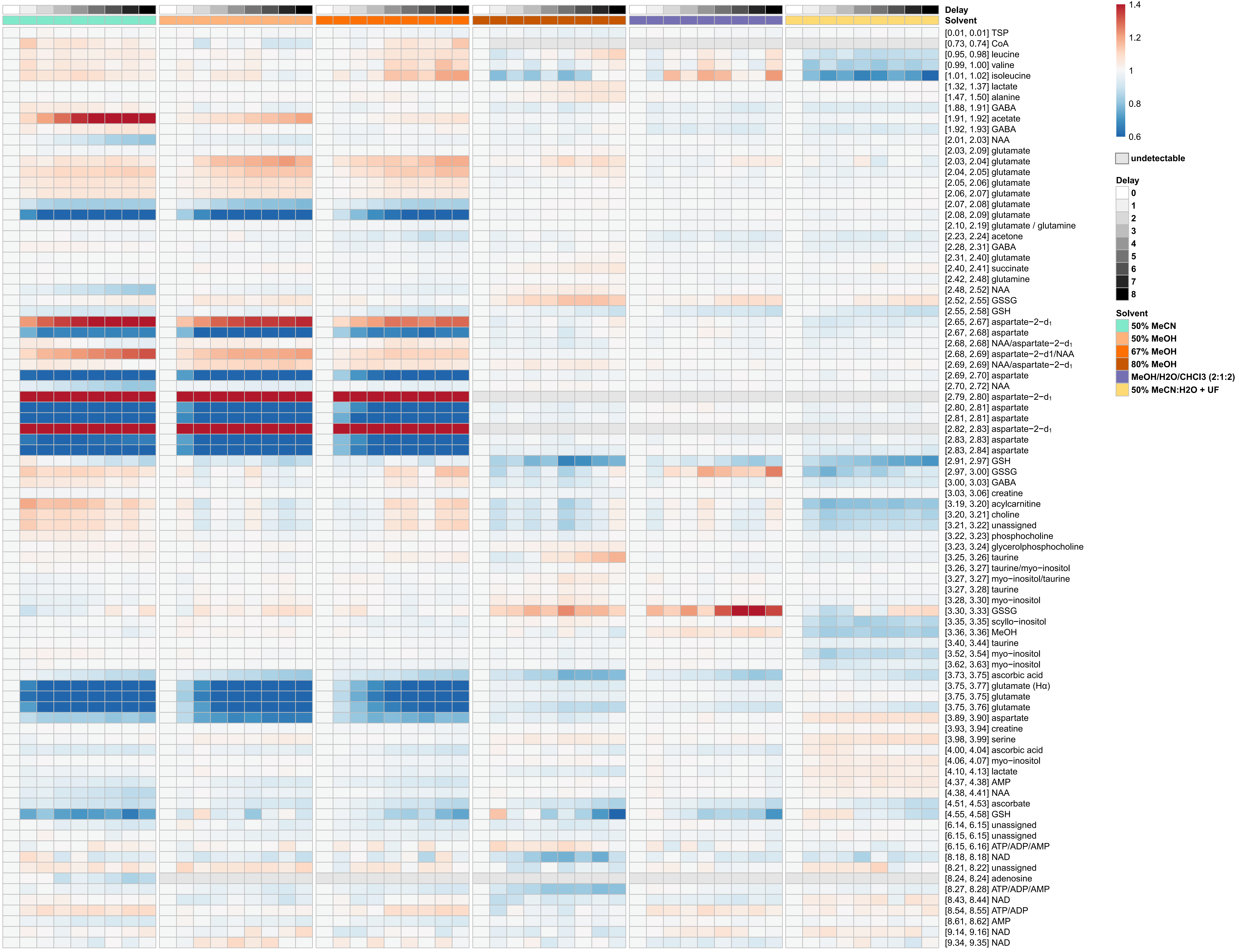

Figure S3. Heatmap of NMR resonance percentage changes for metabolites across extraction methods.

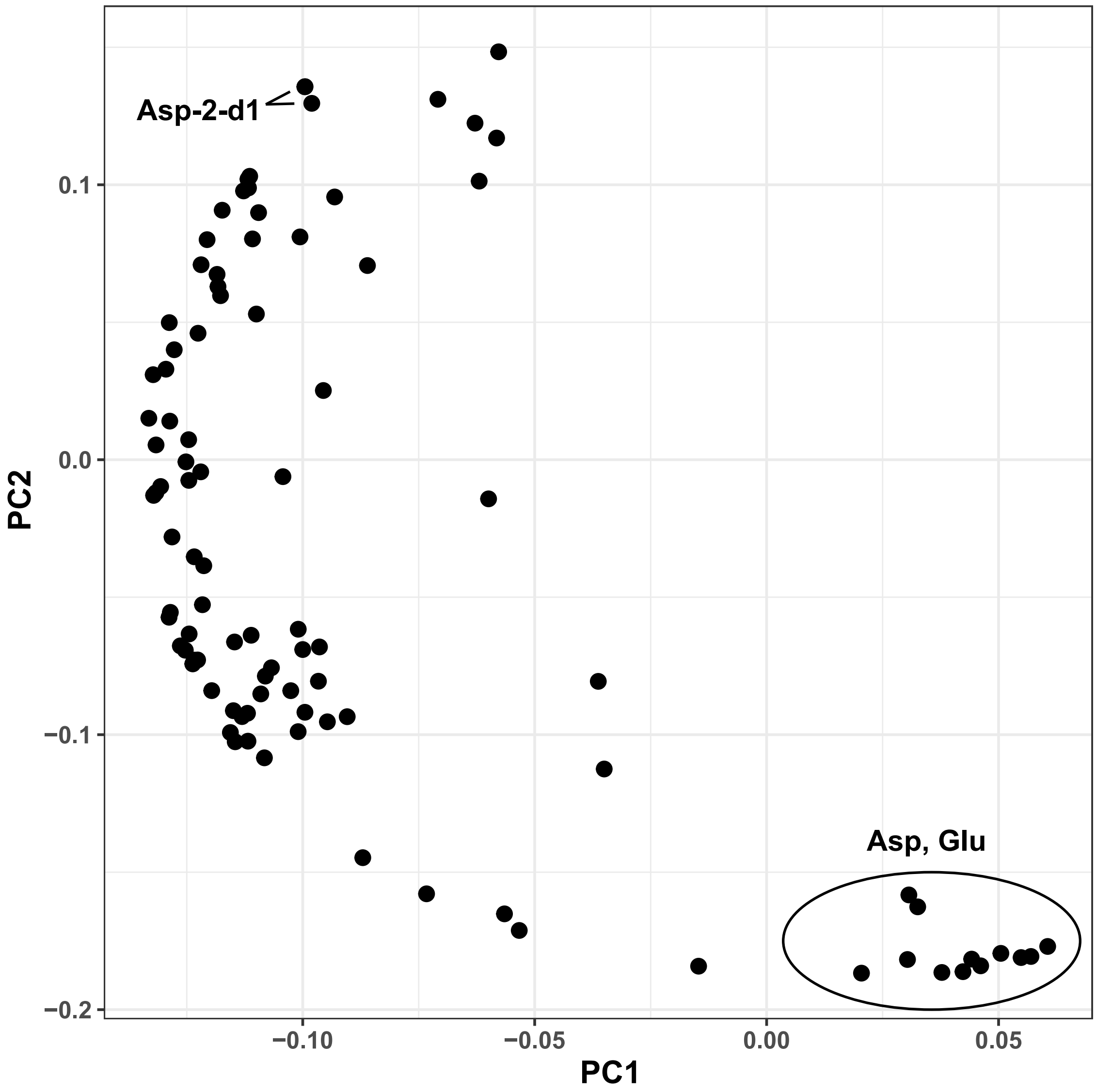

Figure S4. Loadings plot of PCA (corresponding to scores plot on Figure 2D) highlighting the contributions of Asp-2-d1, Asp, and Glu to variation in PC2.

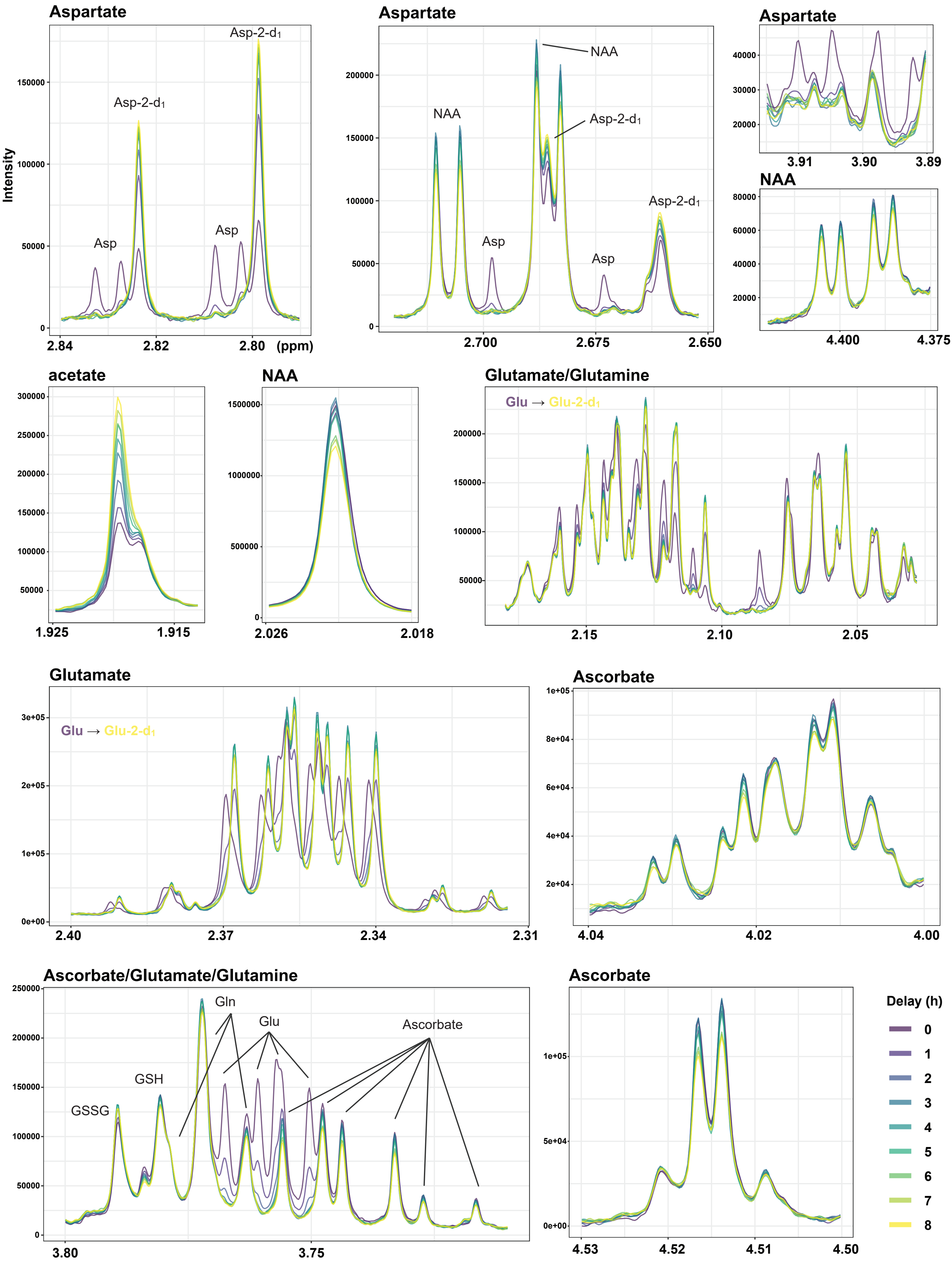
Aspartate
Asp-2-d₁
Asp
Intensity
(ppm)
NAA
Aspartate
NAA
acetate
NAA
Glutamate/Glutamine
Glu → Glu-2-d₁
Glutamate
Glu → Glu-2-d₁
Ascorbate
Ascorbate/Glutamate/Glutamine
Gln
Glu
Ascorbate
GSH
GSSG
Ascorbate
Delay (h)
0
1
2
3
4
5
6
7
8

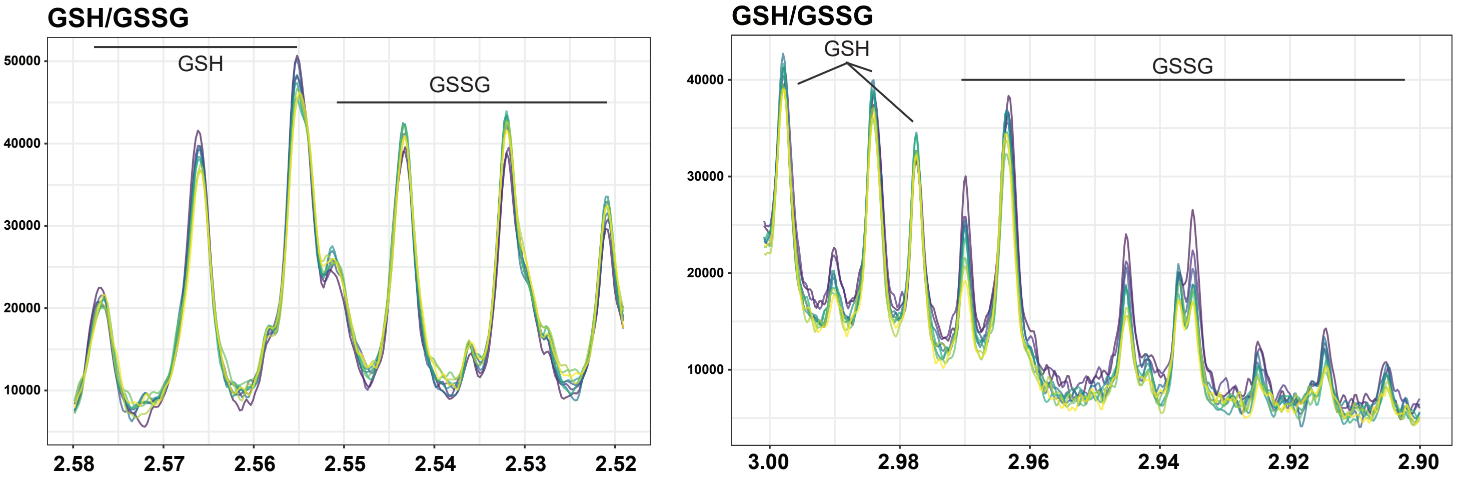

Figure S5. NMR spectra illustrating changes in unstable resonances over time delays in NMR measurement in a 50% MeCN brain sample.

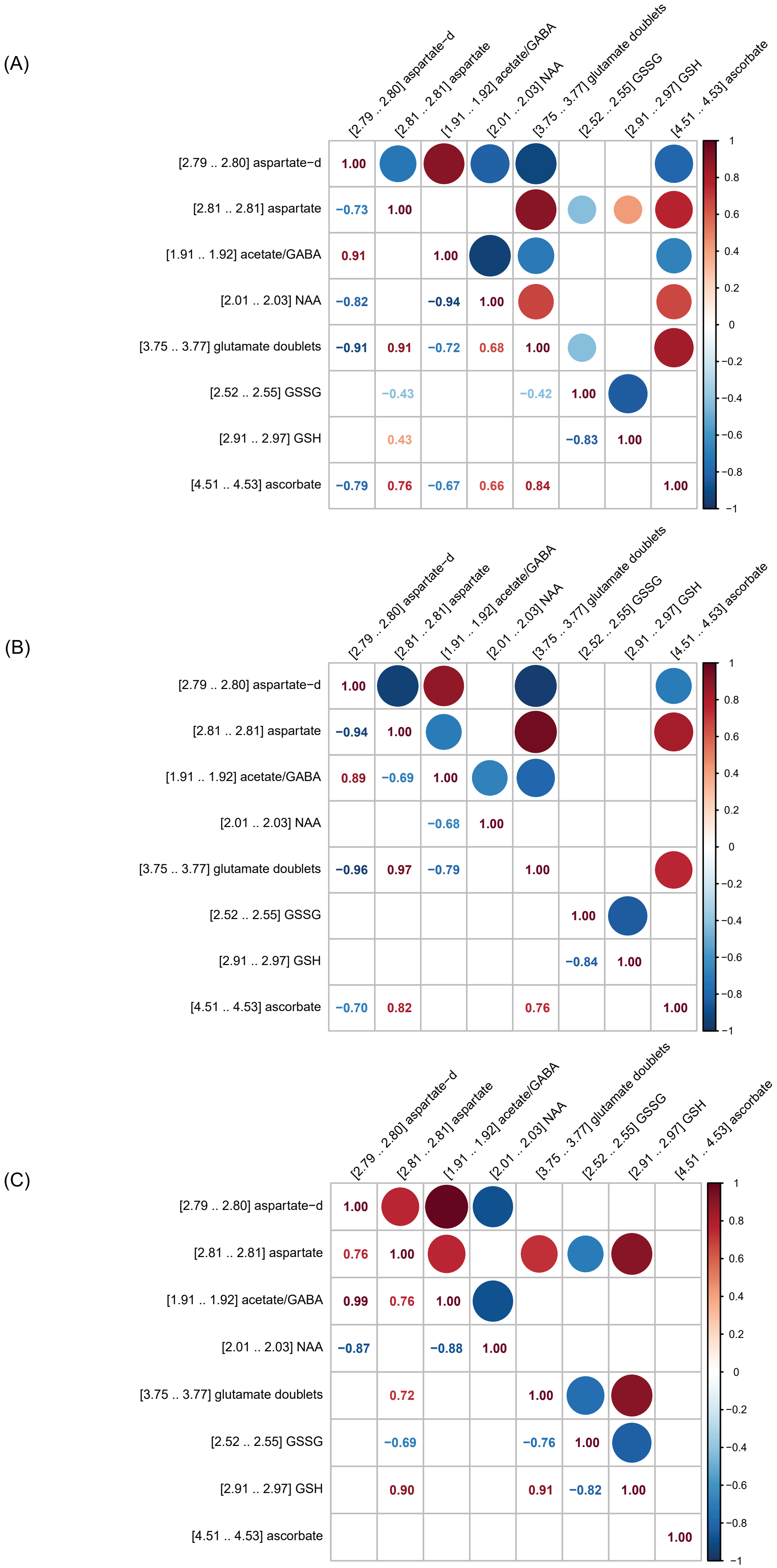

Figure S6. Pearson correlation of unstable metabolites in 50% MeCN samples. Pearson correlation coefficient r is visualised with different sized circles in the upper triangular of the correlation matrix, and the r values are shown in the lower triangular. P values were adjusted with the false discovery rate. The plot includes only significant correlations. (A) 0–8-hour samples. (B) 0–3-hour samples (C) 4-8-hour samples.

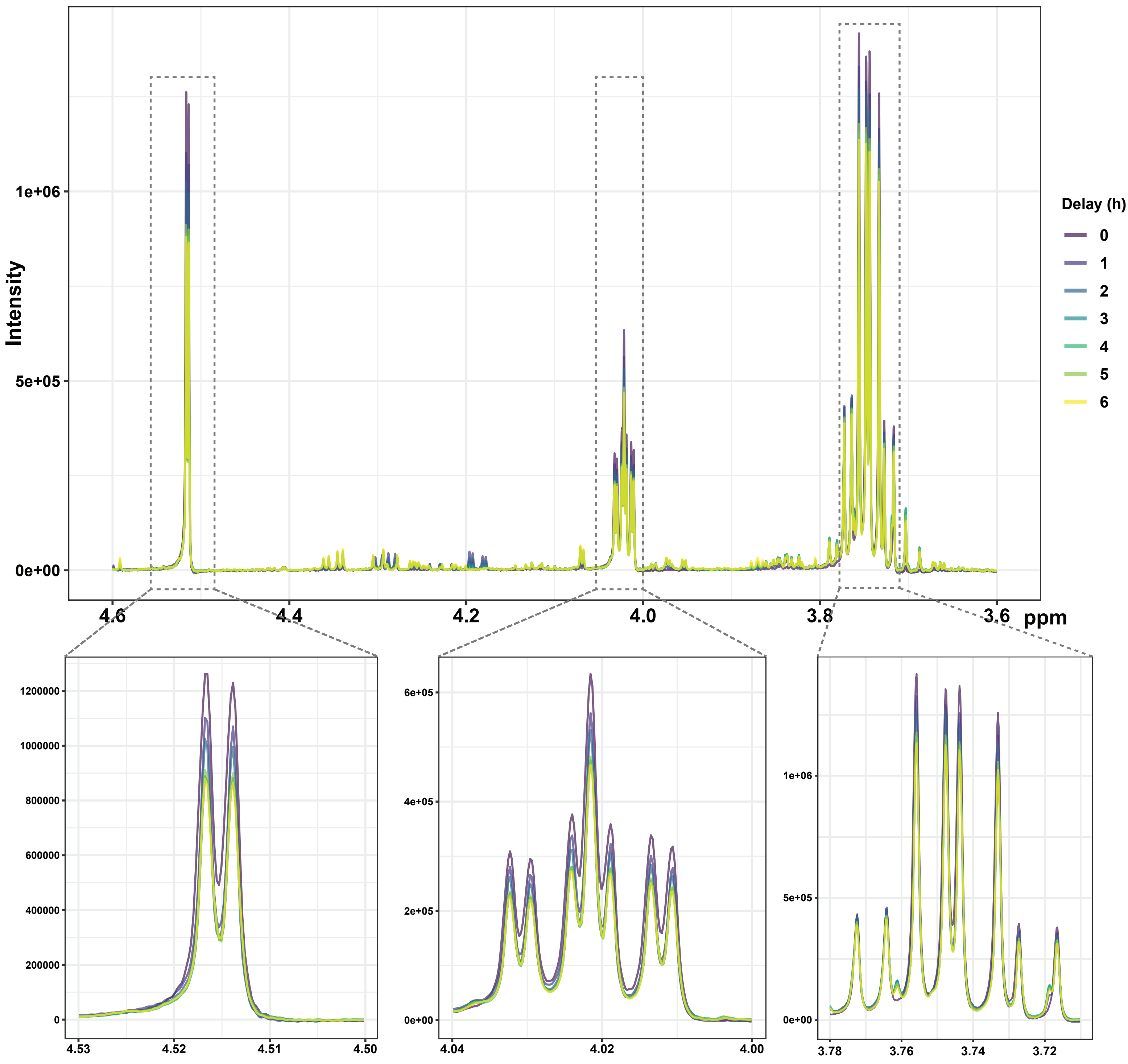

Figure S7. NMR spectra illustrating the reduction in ascorbate signals over time in an ascorbic acid standard sample (in phosphate buffer pH = 7.4) during NMR Measurement.

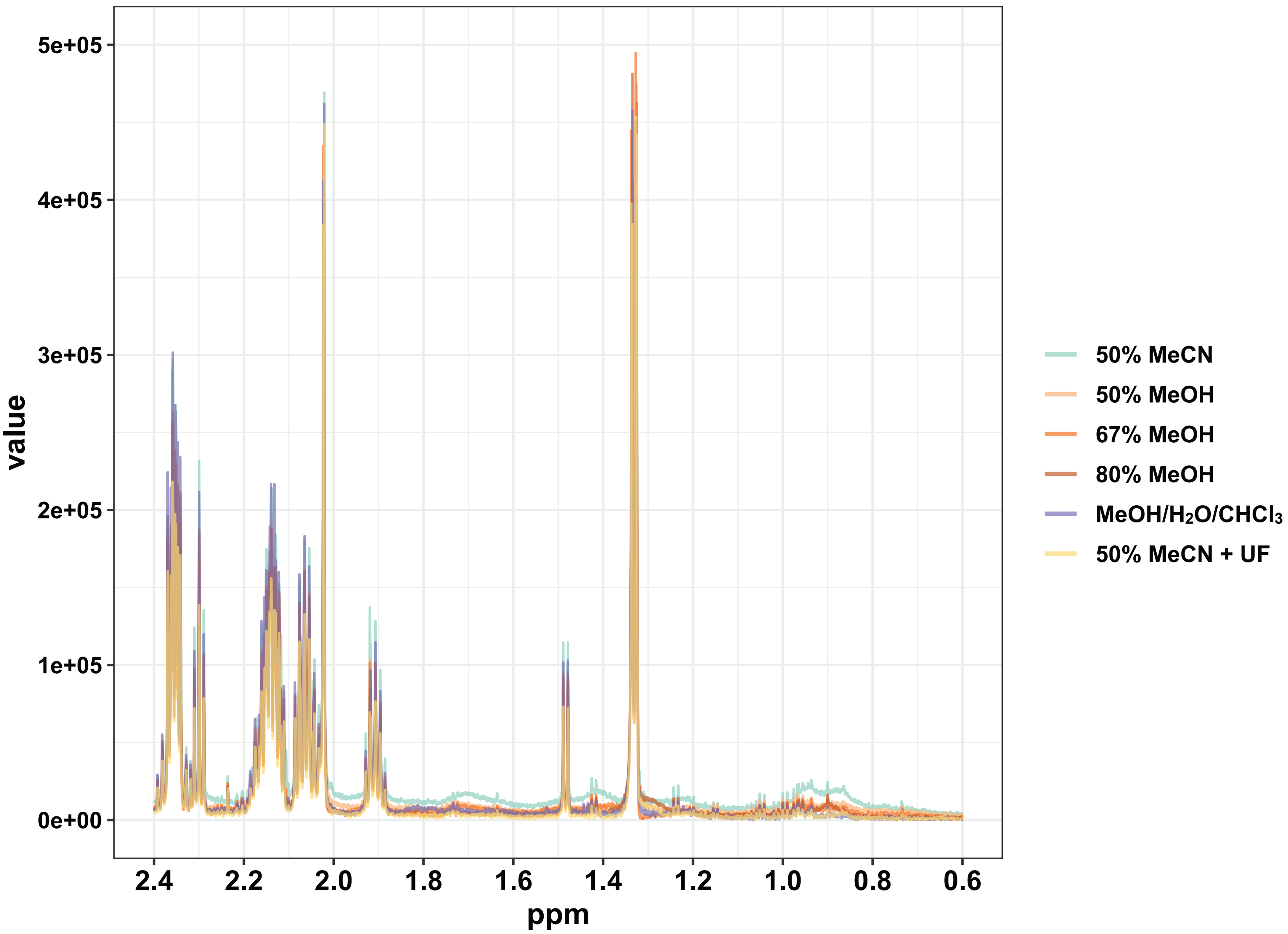

Figure S8. Enhanced baseline uniformity at 0.8 – 1.0 ppm, 1.2 – 1.5 ppm, 1.6 – 1.8 ppm in 50% MeCN samples with ultra-filtration (yellow) in contrast to 50% MeCN samples (green).

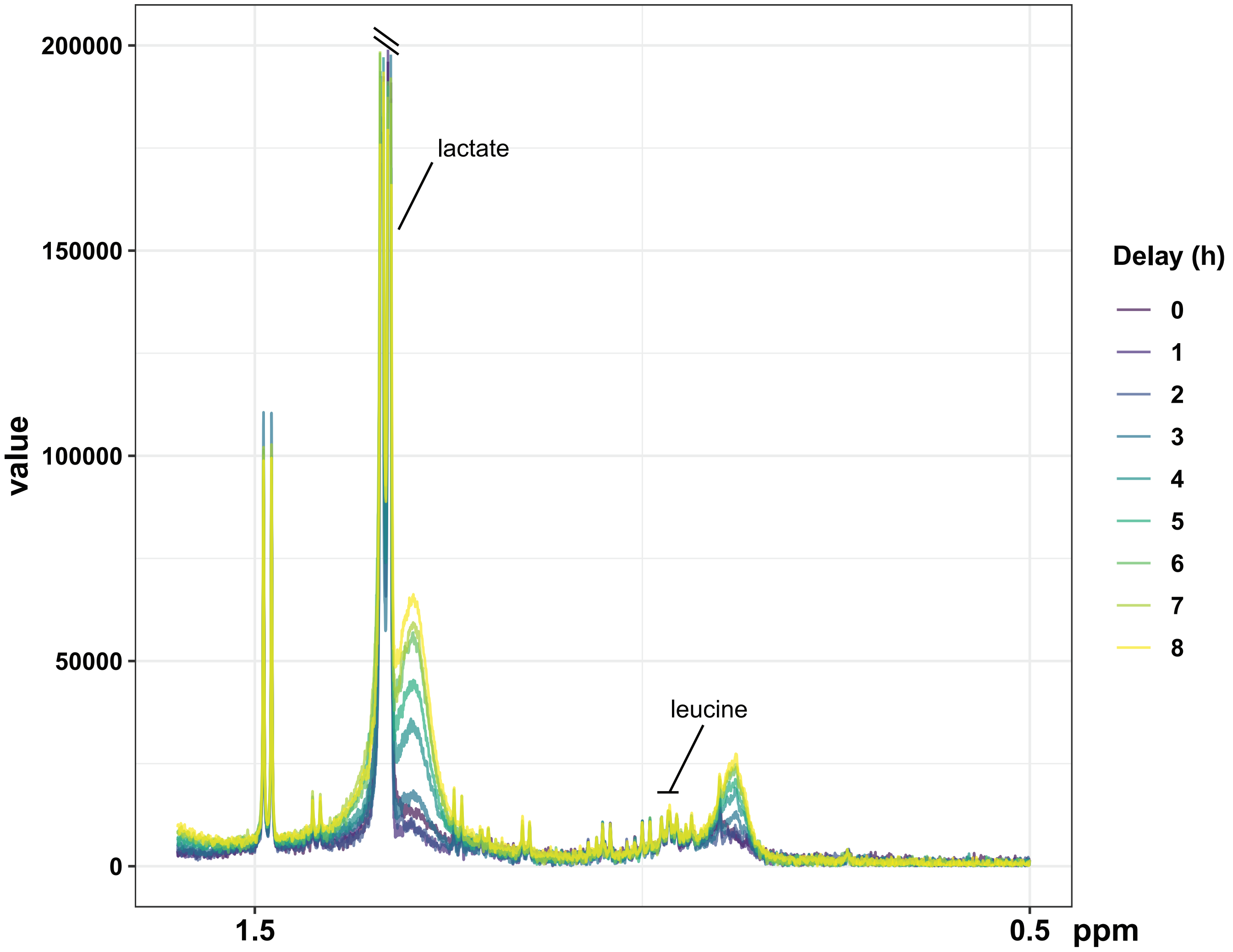

Figure S9. NMR spectra illustrating the increasing broad signals over time at 0.85 – 0.90 ppm and 1.25 – 1.35 ppm in an 80% MeOH brain extract during NMR measurement.

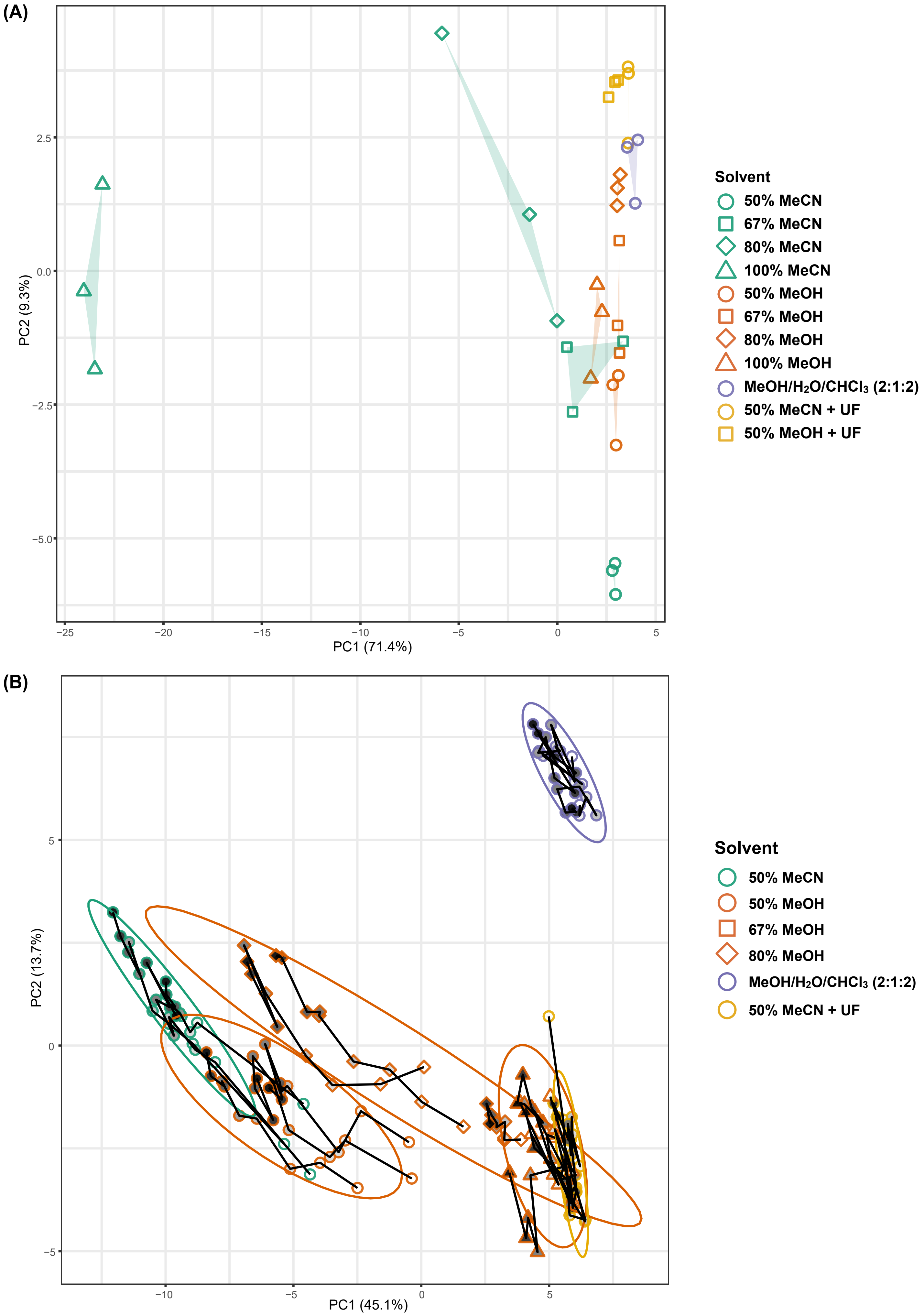

Figure S10. Sum normalised method reduced the impact of variations in overall sample concentration. This is compared to Figure 2D and 3A, where absolute values were used for PCA. (A) PCA scores plot of the brain metabolic profiles from different extraction methods with no delay in NMR measurement. (B) PCA scores plot of brain metabolic profiles from six extraction methods over a time range from 0 to 8 hours of delay in NMR measurement.
